# Integrating multivariate resting‐state fMRI features to localize epileptic networks in common childhood epilepsy

**DOI:** 10.1002/epi.70243

**Published:** 2026-05-06

**Authors:** Qirui Zhang, Zixuan Zhang, Yvzhuo Li, Yan He, Fang Yang, Jacqueline Kim, Joseph I. Tracy, Guangming Lu, Zhiqiang Zhang

**Affiliations:** ^1^ Department of Diagnostic Radiology Jinling Hospital, Medical School of Nanjing University Nanjing Jiangsu China; ^2^ Farber Institute for Neuroscience, Department of Neurology Thomas Jefferson University Philadelphia Pennsylvania USA; ^3^ Department of Neurology Children's Hospital of Nanjing Medical University Nanjing China; ^4^ Department of Neurology Jinling Hospital, Medical School of Nanjing University Nanjing Jiangsu China

**Keywords:** absence epilepsy, BECTS, functional connectivity, latent variable modeling, Rolandic epilepsy

## Abstract

**Objective:**

Accurate localization of epileptic activity remains challenging when interictal discharges are absent or sparse. Although resting‐state functional MRI (rs‐fMRI) is noninvasive, individual rs‐fMRI metrics provide inconsistent and incomplete localization. We aimed to determine whether multivariate integration of rs‐fMRI features could robustly identify syndrome‐specific epileptic activity in childhood epilepsy.

**Methods:**

Using simultaneous EEG‐fMRI, we studied children with self‐limited epilepsy with centrotemporal spikes (SeLECTS, *n* = 60) and childhood absence epilepsy (CAE, *n* = 30) alongside typically developing controls (*n* = 108). Forty‐two rs‐fMRI metrics spanning amplitude, connectivity, temporal dynamics, and directional measures were computed for each session. Individual abnormality maps were also generated relative to controls. Partial least squares (PLS) regression was then applied to identify a latent component (PLS1) that maximally covaried with syndrome‐specific epileptic activation patterns derived from EEG‐fMRI. Spatial correspondence, localization accuracy, non‐discharge session sensitivity, and classification performance were evaluated.

**Results:**

PLS1 showed strong spatial correspondence with EEG‐fMRI–defined epileptic activation patterns in both SeLECTS (rolandic cortex) and CAE (thalamocortical network) (both spin‐test *r* = .68, *p* < .001). PLS1 showed superior localization performance compared with most single‐metric rs‐fMRI measures and reached EEG‐fMRI–level localization accuracy. Notably, PLS1 detected graded, syndrome‐specific abnormalities during non‐discharge sessions and distinguished discharge, no‐discharge, and control states, an effect that has not been observed with individual rs‐fMRI metrics. In classification analyses, PLS1 differentiated CAE from SeLECTS with high accuracy (AUC = .79), performing comparably to the EEG‐fMRI–based classification.

**Significance:**

Multivariate integration of rs‐fMRI features using a template‐guided PLS framework enables sensitive and syndrome‐specific detection of epileptic activity, even in the absence of overt discharges. This approach provides a clinically translatable strategy for noninvasive epilepsy network mapping.

**Key points**

- A template‐guided partial least squares (PLS) framework integrates multivariate resting‐state functional MRI (rs‐fMRI) to map syndrome‐specific epileptic activity in epilepsy.
- This approach identified syndrome‐specific epileptogenic patterns: rolandic cortex in children with self‐limited epilepsy with centrotemporal spikes (SeLECTS) and thalamus in childhood absence epilepsy (CAE).
- Epilepsy‐related abnormalities were detectable even without overt discharges, providing a clinically translatable network mapping strategy.

## 1 INTRODUCTION

Localizing clinically meaningful epileptogenic regions and networks remains a central challenge in epilepsy evaluation.^1^ Simultaneous electroencephalography–functional MRI (EEG‐fMRI) provides a powerful noninvasive approach by linking interictal epileptiform discharges to blood‐oxygen level–dependent (BOLD) responses and by combining the temporal sensitivity of EEG with the spatial coverage of fMRI. EEG‐fMRI has demonstrated translational potential across multiple epilepsy contexts,^2^, ^3^ including MRI‐negative focal cortical dysplasia,^4^ refractory epilepsy,^5^, ^6^, ^7^, ^8^, ^9^, ^10^ and childhood epilepsy syndromes.^2^, ^11^ However, its practical deployment is constrained by technical complexity, variability in hemodynamic responses,^12^ and the fundamental dependence on capturing a sufficient number of discharges during scanning.^13^ Recent evidence further indicates that only ~35% of patients yield clinically meaningful localization from EEG‐fMRI,^10^ limiting its sensitivity and feasibility in routine workflows.

Resting‐state fMRI (rs‐fMRI) offers a more accessible alternative. It requires no additional equipment and can be acquired as part of standard clinical imaging. A growing literature suggests that rs‐fMRI metrics can reveal epilepsy‐related abnormalities and may inform presurgical planning. Reported markers span local amplitude measures (e.g., amplitude of low‐frequency fluctuation [ALFF]/fractional ALFF [fALFF]),^14^, ^15^, ^16^, ^17^ regional synchrony (ReHo),^18^, ^19^ graph‐based centrality/connectivity measures (e.g., degree centrality, functional connectivity density),^14^, ^18^ and directed/temporal measures (e.g., Granger causality density and resting‐state lag analyses).^20^, ^21^ These studies collectively support that epilepsy is reflected in distributed functional alterations that are measurable at rest.

Despite this evidence, most rs‐fMRI approaches remain univariate, relying on one (or a few) metric at a time, and often yield spatially incomplete or inconsistent localization. Even within the same syndrome, different rs‐fMRI metrics can highlight partially overlapping but non‐identical patterns, reflecting the noisy and interdependent nature of BOLD‐derived measures.^15^, ^18^, ^22^, ^23^, ^24^, ^25^, ^26^ This limitation creates a practical barrier for individual‐level inference: it is often unclear which metric best localizes clinically relevant epileptic activity in a given patient or session. A complementary strategy would integrate multiple rs‐fMRI features, leveraging the idea that small and noisy effects across individual metrics can be reduced when combined in a principled multivariate framework.^18^, ^27^, ^28^

In the present study, we test a template‐guided, multivariate approach in two of the most common childhood epilepsy syndromes: self‐limited epilepsy with centrotemporal spikes (SeLECTS, or rolandic epilepsy) and childhood absence epilepsy (CAE), which represent prevalent focal and generalized childhood epilepsies,^29^ respectively. Both syndromes have characteristic electrophysiological signatures^2^ (centrotemporal spikes in SeLECTS; 3 Hz generalized spike–wave discharges in CAE) that enable robust estimation of syndrome‐specific epileptic activation patterns using EEG‐fMRI and, critically, allow evaluation of rs‐fMRI–based detection in both discharge and no‐discharge sessions.

We propose a partial least squares (PLS) regression^30^, ^31^ framework that integrates a broad set of rs‐fMRI metrics to derive a single latent component (PLS1) that maximally covaries with a syndrome‐specific EEG‐fMRI epileptic activation template. Concretely, we compute individual‐session abnormality maps for each rs‐fMRI metric relative to typically developing control (TDC) and then use PLS to identify the weighted combination of these abnormalities that best expresses the EEG‐fMRI–defined epileptic activity pattern. We evaluate this approach along three clinically relevant axes: (1) spatial correspondence and localization accuracy relative to EEG‐fMRI templates, (2) sensitivity to graded epileptic activity during no‐discharge sessions, and (3) diagnostic utility for classification of epilepsy syndrome and patient–control status.

## 2 MATERIALS AND METHODS

### 2.1 Participants

Sixty children with SeLECTS (36 boys and 24 girls, age 9.13 ± 2.10 years) and 30 children with CAE (13 boys and 17 girls, age 9.75 ± 3.48 years) were recruited from Jinling hospital. All patients were diagnosed independently by two certified neurologists (Y.H. and F.Y.) with extensive expertise in pediatric epilepsy, following the diagnostic criteria of the International League Against Epilepsy (ILAE).^29^ Exclusion criteria were: (1) age younger than 5 years or older than 18 years, and (2) the presence of structural abnormalities on routine MRI. In addition, 108 TDC (62 boys and 48 girls; mean age 9.42 ± 2.48 years) were recruited as controls from a local primary school. None of the control participants had a history of neurological or psychiatric disorders, and none were taking any medications at the time of enrollment.

This research was approved by the medical ethics committee in Jinling Hospital, Nanjing University School of Medicine, and written informed consent was obtained from the guardian of each participating child.

### 2.2 Data acquisition

All patients underwent simultaneous EEG‐fMRI data acquisition on a 32‐channel MRI‐compatible EEG (Brain Product, Munich, Germany, 5 kHz sampling rate) and a 3 T Siemens TimTrio, MRI scanner (Erlangen, Germany). Each patient underwent 2–3 sessions of functional MRI scan with a repetition time (TR) of 2 s, with each session consisting of 500 volumes, along with high‐resolution structural MRI data. For the TDC group, only 250 volumes rs‐fMRI data were acquired.

To ensure comparability across groups and to increase the number of analyzable observations, fMRI data were segmented into one or more sessions of 250 consecutive volumes, allowing each session to be categorized as discharge or no‐discharge based on concurrent EEG recordings.

We excluded participants with poor T1w quality and fMRI sessions with large head motion (participants level: 4 SeLECTS, 3 CAE, and 8 TDC). The MRI sequence details and quantity control of image data are detailed in Figure S1.

### 2.3 Data processing

EEG data were processed offline to remove gradient and ballistocardiogram artifacts using Brain Vision Analyzer 2.0 (Brain Products, Munich, Germany). Interictal epileptiform discharges were identified on the artifact‐corrected EEG by an experienced electroencephalographer and an epileptologist.

Among the patients, 45 children with SeLECTS exhibited at least one centrotemporal spike (CTS), and 18 children with CAE exhibited at least one 3‐Hz generalized spike‐and‐wave discharge (GSWD) during EEG‐fMRI acquisition. Based on the presence or absence of epileptiform discharges within each fMRI session, patient data were further categorized into discharge and no‐discharge sessions. Specifically, SeLECTS sessions were divided into SeLECTS‐CTS (117 sessions) and SeLECTS‐no‐CTS (65 sessions), whereas CAE sessions were divided into CAE‐GSWD (44 sessions) and CAE‐no‐GSWD (43 sessions).

#### 2.3.1 fMRI preprocessing

Functional MRI data were preprocessed using the Statistical Parametric Mapping based (SPM 12; http://www.fil.ion.ucl.ac.uk/spm) DPARSF toolbox (DPARSF_V2.3; www.restfmri.net).^32^

Preprocessing steps included: (1) discarding the first 10 volumes to allow for signal equilibration; (2) slice‐timing correction; (3) head motion correction; (4) co‐registration to the corresponding three‐dimensional T1‐weighted anatomic image, followed by spatial normalization to the Montreal Neurological Institute (MNI)152 template using a 12‐parameter affine transformation and resampling to a resolution of 3 × 3 × 3 mm^3^; (5) spatial smoothing with an isotropic Gaussian kernel of 8 mm full width at half maximum (FWHM); and (6) nuisance regression to remove the effects of head motion parameters, white matter signal, and cerebrospinal fluid signal.

To characterize frequency‐dependent properties of BOLD fluctuations, data were band‐pass filtered not only within the conventional frequency range (.01–.10 Hz) but also subdivided into four additional narrow frequency bands: .01–.027 Hz, .027–.073 Hz, .073–.198 Hz, and .198–.25 Hz.^33^ Together with the conventional band, these five frequency ranges were used to compute frequency‐specific resting‐state fMRI metrics, allowing more detailed characterization of BOLD signal fluctuations across multiple temporal scales.

#### 2.3.2 EEG‐fMRI analysis

Epileptic activity–related fMRI activation associated with CTSs or GSWDs was evaluated using a general linear model, implemented in SPM12. Each CTS or GSWD was modeled as an event and convolved with the canonical hemodynamic response function. Head motion realignment parameters were included as nuisance covariates.

**FIGURE 1:**
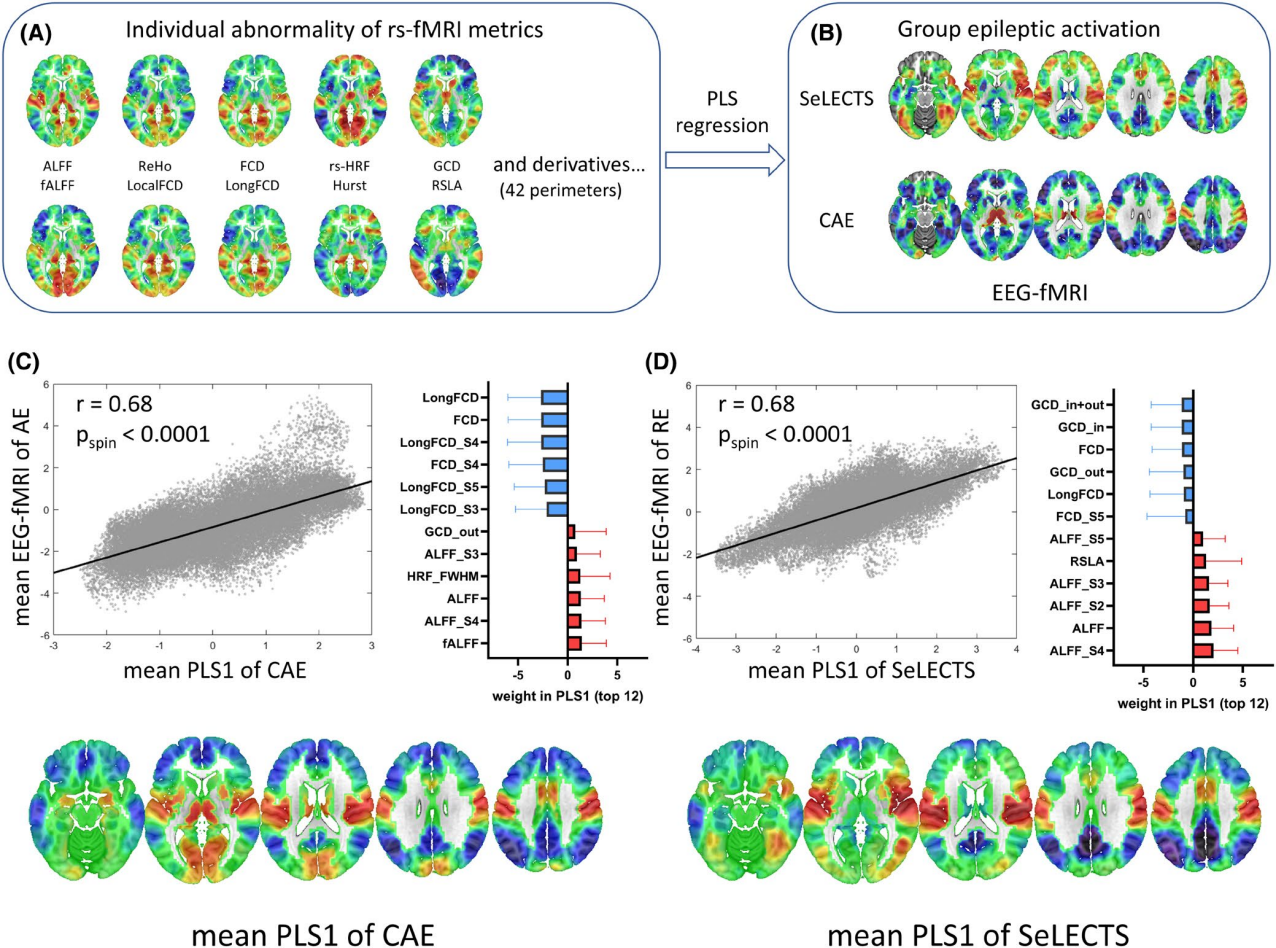
Derivation of the partial least squares‐based epileptic activity component (PLS1). (A) Individual abnormality maps of resting‐state fMRI (rs‐fMRI) metrics from a representative session. (B) Group‐level epileptic activation map derived from simultaneous electroencephalography–functional MRI (EEG‐fMRI), obtained using a one‐sample *t* test of interictal epileptiform discharge–related BOLD responses within each epilepsy syndrome (self‐limited epilepsy with centrotemporal spikes [SeLECTS] or childhood absence epilepsy [CAE]). Partial least squares regression was performed between the individual rs‐fMRI abnormality maps (A) and the corresponding group epileptic activation map (B) to extract the first latent component (PLS1), representing the rs‐fMRI pattern most strongly associated with epileptic activity. (C, D) Spatial correspondence between the mean group epileptic activation maps (as shown in B) and the mean PLS1 maps for CAE (C) and SeLECTS (D), visualized using voxel‐wise spatial scatter plots. The corresponding PLS1 feature weights for each rs‐fMRI metric are displayed below each scatter plot, and the group‐averaged PLS1 spatial maps for CAE and SeLECTS are shown at the bottom. Error bars indicate standard deviation across sessions.

For each epilepsy syndrome, group‐level epileptic activation maps were generated using a second‐level random‐effects analysis (one‐sample *t*‐test) applied to individual CTS‐ or GSWD‐related contrast maps (Figure 1B). This analysis identified brain regions showing significant BOLD signal changes associated with epileptic discharges at the group level.

#### 2.3.3 Resting‐state fMRI analysis

To comprehensively assess the capacity of resting‐state fMRI to localize epileptic activity, a broad set of regional rs‐fMRI metrics was computed. These included amplitude of low‐frequency fluctuation (or ALFF),^34^ fractional ALFF (or fALFF),^35^ regional homogeneity (or ReHo),^36^ Hurst exponent (Hurst),^37^ degree centrality (DC),^38^ and long‐ and short‐range functional connectivity density (FCD).^39^ To capture physiological information present at different temporal scales, all applicable metrics were additionally computed within the four narrow frequency bands we described.

In addition, directed and temporal measures of brain activity were calculated, including Granger causality density (GCD),^40^ resting‐state lag analysis (RSLA),^41^ and hemodynamic response function (HRF)–related parameters.^42^ Deconvolved HRF‐based ALFF, fALFF, and DC measures were also derived.

Definitions and calculation of all rs‐fMRI parameters are provided in Figure S1. In total, 42 rs‐fMRI parameters were obtained for each session. All parameter maps were masked using a gray matter probability mask with a threshold of .2 before subsequent analyses.

### 2.4 Detection of epileptic activity using multivariate rs‐fMRI

#### 2.4.1 Individual abnormality maps of rs‐fMRI metrics

To quantify session‐level abnormalities in rs‐fMRI metrics, voxel‐wise abnormality maps were generated using SPM12 by contrasting each session against the normative distribution defined by the TDC group (Figure 1A). For epilepsy sessions, each map was computed by contrasting that session with the full TDC group. For TDC sessions, a leave‐one‐out approach was applied, in which each control session was contrasted against the remaining TDC sessions. The resulting maps represent voxel‐wise t‐statistics, where each voxel value reflects the standardized deviation (SD) of that session relative to the normative distribution defined by the TDC group.^43^

#### 2.4.2 Multivariate synthesis of epileptic activity using partial least squares regression

To integrate information across multiple resting‐state fMRI (rs‐fMRI) metrics and derive a composite marker of epileptic activity, PLS regression was used to model the relationship between session‐level rs‐fMRI abnormality maps (42 metrics per session; Figure 1A) and syndrome‐specific epileptic activation patterns derived from EEG‐fMRI (Figure 1B). The first latent component of the PLS model (PLS1) was defined as the weighted linear combination of rs‐fMRI metrics that maximized covariance with the epileptic activation pattern, and the corresponding feature weights were retained to quantify the relative contribution of each metric.

Because epileptic activation patterns differ across epilepsy syndromes, separate PLS models were constructed using group‐level epileptic activation maps from SeLECTS and CAE, respectively. For each syndrome‐specific model, the corresponding template was used to compute PLS1 representations for all sessions, including both epilepsy and TDC sessions. As a result, each session was characterized by two syndrome‐specific PLS1 representations, which were interpreted as composite, session‐level indices of epileptic activity.

### 2.5 Evaluation of epileptic activity detection

Epileptic activity exhibits substantial within‐subject variability across recording sessions, and our primary objective was to evaluate session‐level epileptic activity detection rather than between‐syndrome group differences. Therefore, each session was treated as an independent observational unit in subsequent analyses.

#### 2.5.1 Spatial similarity of epileptic activity pattern

We performed spatial correlation analysis between PLS1 and group epileptic activation at the individual and group level. Spin permutation testing, based on spherical rotations of the PLS1 map (5000 times),^44^ was used to test the following hypothesis: PLS1 explains more covariance between group epileptic activation and rs‐fMRI metrics than expected by chance.^45^

#### 2.5.2 Localization ability of epileptic activity

The localization ability of epileptic activity was quantified using the Dice similarity coefficient, which measures spatial overlap between individual‐session maps and the corresponding group epileptic activation map derived from EEG–fMRI. Dice coefficients were computed between individual PLS1 maps and the group epileptic activation map.

Dice coefficients require binary inputs. Consequently, both activation and deactivation maps of PLS1, as well as the group‐level epileptic activation map, were thresholded at the top 5% of voxel‐wise values to generate binary images. The same procedure was applied to individual abnormality maps of representative conventional rs‐fMRI metrics, including ALFF, ReHo, DC, Hurst exponent, GCD (represented by GCD in–out), and HRF (represented by HRF height). Dice coefficients were also calculated for individual EEG‐fMRI activation maps for comparison.

One‐way analysis of variance (ANOVA) followed by post hoc comparisons (*p* < .05, Dunnett corrected) was performed to compare Dice coefficients across imaging metrics.

#### 2.5.3 Localization ability in no‐discharge session

To evaluate whether imaging metrics could detect latent epileptic abnormalities in the absence of overt interictal discharges, we examined localization performance in no‐discharge resting‐state sessions.

First, voxel‐wise individual abnormality maps derived from PLS1 and conventional rs‐fMRI metrics were compared using two‐sample *t*‐tests between discharge sessions and TDC sessions, as well as between no‐discharge sessions and TDC sessions (*p* < .05, uncorrected).

Next, region of interest (ROI)–based analyses were performed to quantify epileptic activity within syndrome‐specific epileptic regions. Individual abnormality values were extracted from PLS1 and rs‐fMRI metrics within predefined epileptic ROIs. For CAE, the epileptic ROI was defined as the significant thalamic region identified in the CAE group epileptic activation map. For SeLECTS, the epileptic ROI was defined as the significant sensorimotor (rolandic) cortical region identified in the SeLECTS group epileptic activation map. One‐way ANOVA followed by post hoc comparisons (*p* < .05, corrected) was conducted to compare discharge sessions, no‐discharge sessions, and TDC sessions.

#### 2.5.4 Multivariate classification of epilepsy syndromes and patient–control status

To evaluate the diagnostic utility of the PLS1, multivariate classification analyses were performed at the session level. Features were extracted from individual‐session abnormality maps of PLS1 or, for comparison, from individual EEG‐fMRI activation maps using the Automated Anatomical Labeling (AAL) atlas, yielding 90 regional features per session.

Two classification tasks were constructed with different session inclusion criteria. First, for differential diagnosis between CAE and SeLECTS, only epilepsy sessions containing interictal epileptiform discharges during EEG‐fMRI acquisition were included. In this task, classification performance based on PLS1 was directly compared with that based on EEG‐fMRI activation maps. Second, for discrimination between epilepsy and TDC, all available sessions were included, irrespective of whether epileptic discharges were present. In this task, classification was performed using PLS1‐derived features only, as EEG–fMRI activation maps are not defined for no‐discharge or control sessions.

Classification analyses were performed using a subject‐wise grouped nested cross‐validation framework. All sessions from the same participant were assigned to the same fold, such that training and test partitions were independent at the subject level. Classification models were trained using elastic‐net logistic regression with fivefold cross‐validation. The model combines L1 and L2 penalties, enabling both embedded feature selection and coefficient stabilization. Model performance was evaluated using accuracy, sensitivity, specificity, and the area under the receiver‐operating characteristic (ROC) curve (AUC).

## 3 RESULTS

### 3.1 Demographic and clinical characteristics

One hundred eighty‐two SeLECTS sessions (56 participants), 87 CAE sessions (27 participants), and 100 TDC were included for statistical analysis. The details of the demographics and clinical data of all participants are shown in Table 1.

**TABLE 1:** Sample demographic and clinical characteristics.

	SeLECTS		CAE		TDC	*p‐*value
No. of subjects	56		27		100	‐
Sex (male/female)	33/23		12/15		55/45	.46^a^
Age, y Year	9.08 ± 2.12		7.66 ± 2.64		9.48 ± 2.49	.003^b^
Seizure duration, mo	22.72 ± 21.85		33.86 ± 48.66		–	–
No. of sessions	182		87		100	–
Sub‐group	CTS	No‐CTS	GSWD	No‐GSWD	TDC	–
No. of sessions	117	65	44	43	100	–
Head motion	.14 ± .06	.14 ± .06	.14 ± .06	.12 ± .06	.13 ± .05	.34^b^
Discharge times	21.14 ± 19.66	–	6.79 ± 6.56	–	–	–

*Note*: Continuous variables are presented in mean ± SD.

Abbreviations: CAE: Childhood absence epilepsy; CTS: centrotemporal spike; GSWD: generalized spike‐and‐wave discharge; TDC: Typically developing control; SeLECTS: Self‐limited epilepsy with centrotemporal spikes.

^a^ Chi‐square test.

^b^ One‐way ANOVA.

### 3.2 Spatial pattern of epileptic activity detection

PLS1 exhibited strong spatial correspondence with syndrome‐specific epileptic activation patterns derived from EEG‐fMRI. Specifically, the mean PLS1 maps for CAE showed a robust spatial correlation with the CAE group epileptic activation map (*r* = .68, *p*_spin_ <.001), and a similarly strong correspondence was observed for SeLECTS (*r* = .68, *p*_spin_ <.001, Figure 1).

In CAE, the PLS1 spatial pattern was characterized by prominent activation of the thalamus and bilateral sensorimotor cortices, consistent with the canonical thalamocortical network underlying absence seizures. In contrast, PLS1 in SeLECTS was primarily localized to the rolandic region, reflecting the focal cortical nature of centrotemporal epileptic activity. In both syndromes, deactivation was consistently observed within the default mode network.

Analysis of PLS1 feature weights further revealed syndrome‐specific contributions of rs‐fMRI metrics. In CAE, PLS1 was predominantly driven by amplitude‐ and connectivity‐related measures, particularly FCD and ALFF. In SeLECTS, GCD‐related metrics contributed additionally to PLS1, suggesting a greater involvement of directed functional interactions in focal epileptic networks (Figure 1).

### 3.3 Localization ability of epileptic activity

Mean individual‐session abnormality maps of PLS1 exhibited focal activation within syndrome‐specific epileptic regions, most prominently the thalamus in CAE and the rolandic cortex in SeLECTS. In contrast, conventional rs‐fMRI metrics were able to detect activity within the relevant regions to varying extents but typically showed either widespread, nonspecific abnormalities or insufficient focal activation within the epileptogenic zones.

Quantitative assessment using Dice similarity coefficients confirmed the superior localization performance of PLS1. In CAE sessions, Dice coefficients of PLS1 were significantly higher than those of all conventional rs‐fMRI metrics, although they remained lower than those obtained from individual EEG‐fMRI activation maps. In SeLECTS sessions, Dice coefficients of PLS1 were significantly higher than those of most rs‐fMRI metrics, including ReHo, DC, Hurst exponent, GCD, and HRF‐related measures, and did not differ significantly from individual EEG‐fMRI.

**FIGURE 2:**
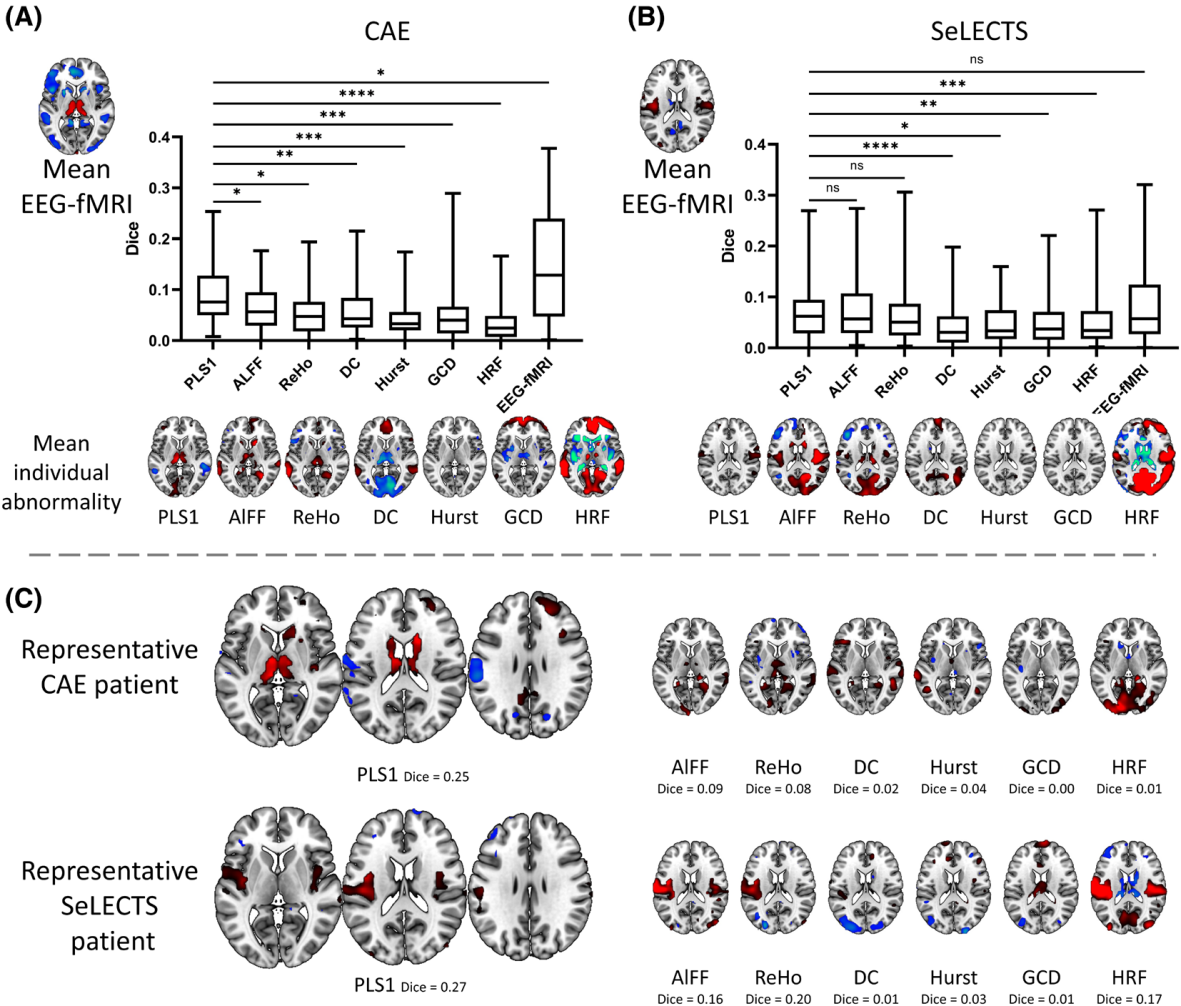
Localization performance of epileptic activity across imaging metrics. Localization accuracy was quantified using Dice similarity coefficients between individual‐session abnormality maps and the corresponding group epileptic activation map derived from simultaneous electroencephalography–functional MRI (EEG–fMRI). Dice coefficients were calculated for the partial least squares‐based epileptic activity component (PLS1), representative conventional resting‐state fMRI metrics, individual EEG–fMRI activation maps, and the group‐level EEG–fMRI activation map. (A, B) Comparison of epileptic activity localization performance (Dice coefficients) across imaging metrics in (A) Childhood absence epilepsy (CAE) and (B) Self‐limited epilepsy with centrotemporal spikes (SeLECTS) sessions. Bar plots depict group‐level Dice coefficients, and top 5% of mean spatial maps for each metric are displayed alongside for visual comparison. (C) Representative examples from a CAE session and a SeLECTS session, illustrating top 5% of individual PLS1 maps, selected rs‐fMRI parameter maps, and their corresponding Dice coefficients relative to the group epileptic activation map.

Representative sessions with the highest Dice coefficients are shown for both CAE and SeLECTS, further illustrating the advantage of PLS1 in accurately localizing epileptic activity at the individual‐session level (Figure 2).

### 3.4 Localization ability in no‐discharge session

An effective measure of epileptic activity should be sensitive to weak, latent abnormalities during no‐discharge resting‐state sessions, while still exhibiting stronger expression during discharge periods. PLS1 satisfied this criterion by demonstrating graded, syndrome‐specific activation across discharge, no‐discharge, and control sessions.

At the whole‐brain level, PLS1 maps from discharge sessions showed significantly greater activation within canonical epileptic regions compared with TDC sessions, with the thalamus most prominently involved in CAE and the rolandic region in SeLECTS. Of note, these same regions were also detectable in no‐discharge sessions, albeit with reduced spatial extent and statistical strength.

ROI‐based analyses further revealed a clear stepwise pattern of epileptic activity captured uniquely by PLS1. In CAE, thalamic PLS1 values exhibited a significant monotonic decrease across conditions (discharge sessions > no‐discharge sessions > TDC sessions; *F* = 17.67, *p* < .001). Post hoc comparisons confirmed significant differences between discharge and no‐discharge sessions (adjusted *p* = .041), as well as between no‐discharge sessions and TDC sessions (adjusted *p* = .011).

**FIGURE 3:**
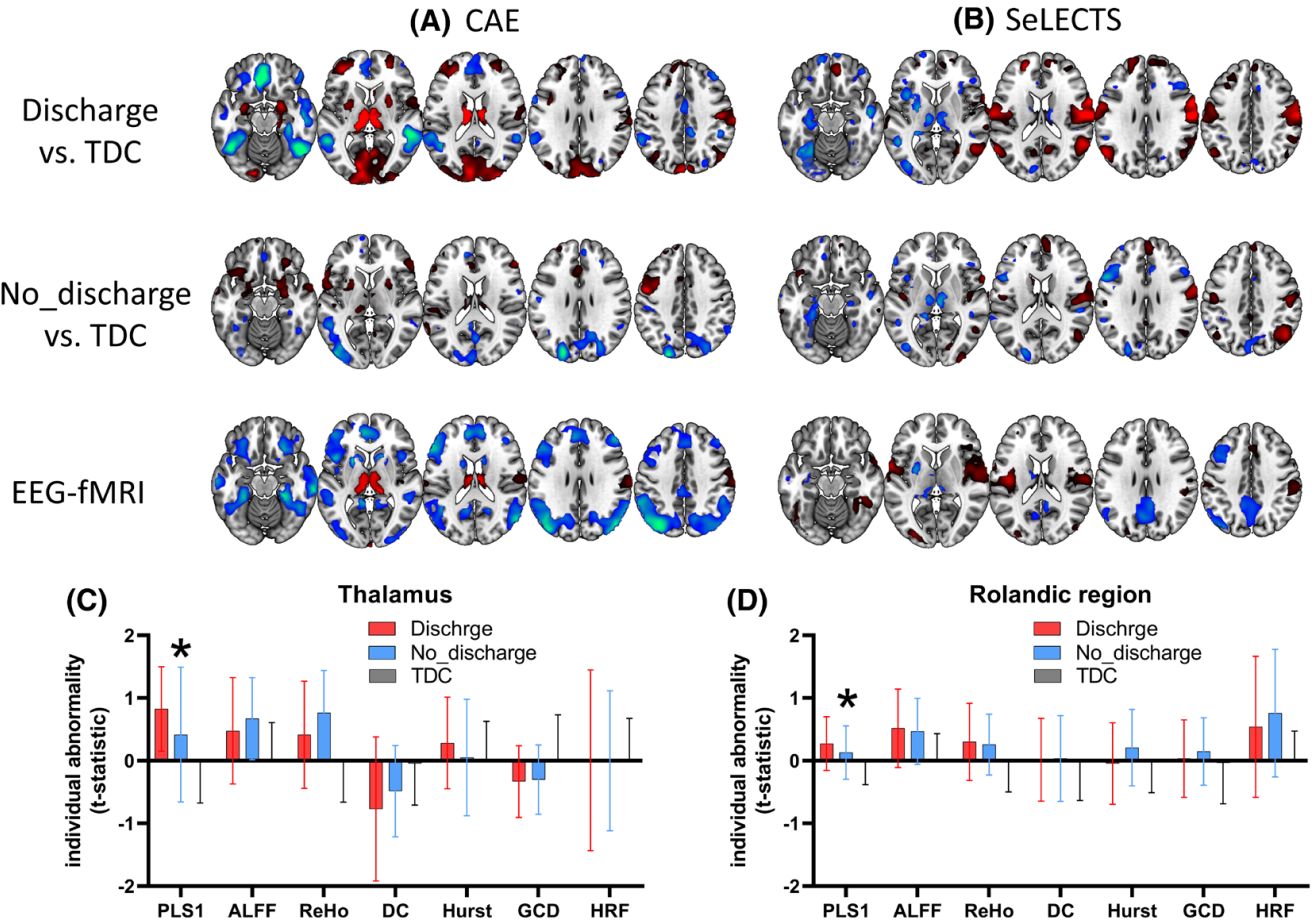
Localization of epileptic activity during no‐discharge resting‐state sessions. To assess whether the partial least squares‐based epileptic activity component (PLS1) can detect latent epileptic abnormalities in the absence of overt interictal discharges, PLS1 maps were compared across epilepsy sessions with discharges, sessions without discharges, and typically developing control (TDC) sessions. (A) Group‐level comparisons of PLS1 maps between childhood absence epilepsy (CAE) discharge sessions and TDC sessions, and between CAE no‐discharge sessions and TDC sessions. (B) Group‐level comparisons of PLS1 maps between self‐limited epilepsy with centrotemporal spikes (SeLECTS) discharge sessions and TDC sessions, and between SeLECTS no‐discharge sessions and TDC sessions. (C) Region of interest analysis in the thalamus, a key epileptic region in CAE. Bar plots illustrate PLS1 values and representative conventional resting‐state fMRI metrics across CAE discharge sessions, CAE no‐discharge sessions, and TDC sessions. (D) Region of interest analysis in the rolandic region, a core epileptic region in SeLECTS. Bar plots show PLS1 values and representative rs‐fMRI metrics across SeLECTS discharge sessions, SeLECTS no‐discharge sessions, and TDC sessions. * indicate metrics exhibiting a statistically significant monotonic decrease across conditions (discharge sessions > no‐discharge sessions > TDC sessions), as assessed by one‐way ANOVA followed by post hoc comparisons.

A similar stepwise trend was observed in the rolandic region for SeLECTS (*F* = 11.95, *p* < .001), with significant differences between discharge and no‐discharge sessions (adjusted *p* = .045) and between no‐discharge sessions and TDC sessions (adjusted *p* = .048). In contrast, none of the conventional rs‐fMRI metrics demonstrated this graded pattern across conditions in either epilepsy syndrome (Figure 3). To further account for the potential influence of multiple sessions from the same subject, we performed a mixed‐effects model analysis as a sensitivity test for the PLS1 results, accounting for repeated observations within subjects. A significant stepwise trend was observed in both CAE (*F* = 6.75, *p* = .002) and SeLECTS (*F* = 7.75, *p* < .001).

### 3.5 Classification ability

PLS1‐based classification demonstrated strong performance in differentiating epilepsy syndromes and epilepsy from control sessions. In the differential diagnosis task, PLS1 accurately distinguished CAE from SeLECTS sessions, with an AUC of .79, performing comparably to the EEG‐fMRI–based classifier (AUC = .77).

Feature‐weight analysis revealed consistent syndrome‐specific patterns across models. In both PLS1‐ and EEG‐fMRI–based classifiers, thalamic regions carried the highest weights for CAE. This convergence suggests that increased thalamic activation represents the principal discriminative feature separating the two syndromes, consistent with the well‐established thalamocortical involvement in absence epilepsy.

In epilepsy–control classification, PLS1 also showed differential efficacy across syndromes. PLS1 distinguished SeLECTS sessions from TDC sessions with an AUC of .67 and CAE sessions from TDC sessions with an AUC of .74. In the SeLECTS vs TDC task, the postcentral gyrus and rolandic operculum contributed most strongly to classification, corresponding to the classical rolandic epileptogenic region,^2^, ^11^, ^19^ whereas in the CAE vs TDC task, the thalamic regions showed the highest feature weights.^46^, ^47^, ^48^

**FIGURE 4:**
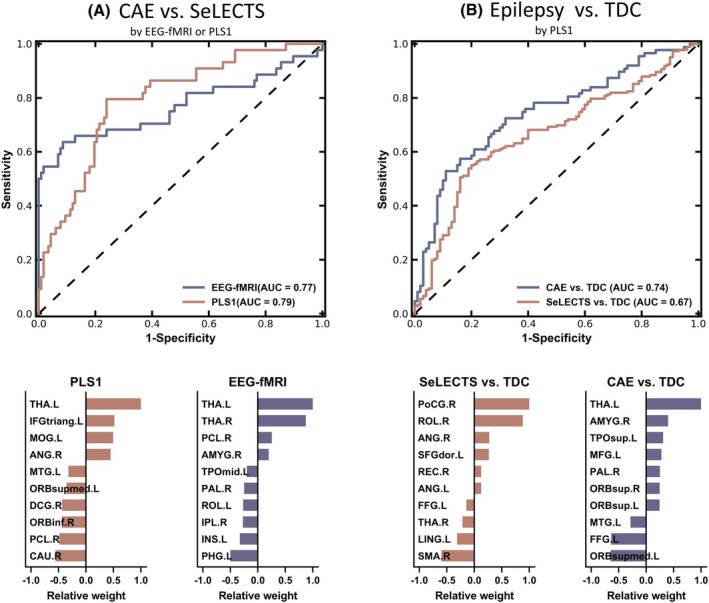
Classification performance of partial least squares‐based epileptic activity component (PLS1)‐based models at the session level. (A) Receiver‐operating characteristic (ROC) curves illustrating the ability of PLS1 to differentiate childhood absence epilepsy (CAE) and self‐limited epilepsy with centrotemporal spikes (SeLECTS) using sessions with epileptic discharges, compared with classification based on simultaneous electroencephalography–functional MRI (EEG‐fMRI). Bar plots below show the corresponding feature weights of each classification model. (B) ROC curves demonstrating the performance of PLS1‐based classification in differentiating CAE sessions from typically developing control (TDC) sessions, and SeLECTS sessions from TDC sessions, using all available sessions. Bar plots below depict the corresponding model feature weights.

Together, these results indicate that PLS1 captures syndrome‐specific and clinically relevant patterns of epileptic activity that support both differential diagnosis between epilepsy syndromes and discrimination between epilepsy and TDC (Figure 4).

## 4 DISCUSSION

In this study, we introduced a novel PLS regression framework that integrates rs‐fMRI metrics to detect and localize epileptic activity in common pediatric epilepsy syndromes. The first latent component (PLS1) successfully identified syndrome‐specific epileptogenic zones—the rolandic cortex in SeLECTS and the thalamus in CAE—demonstrating anatomically precise localization of epileptic networks. PLS1 activation remained evident (albeit reduced) in these regions even during resting‐state periods with no interictal discharges, suggesting that it captures a latent epileptogenic state, not merely transient spike‐related events. Furthermore, PLS1 outperformed most conventional rs‐fMRI metrics in co‐localizing with EEG‐defined epileptic foci (yielding higher Dice similarity) while achieving spatial concordance comparable to gold‐standard EEG‐fMRI maps. This integrated measure distinguished SeLECTS from CAE with performance comparable to EEG‐fMRI–based classification; in addition, PLS1 enabled discrimination between children with epilepsy and TDC. These findings establish PLS1 as a robust, syndrome‐specific marker of epileptic activity in children, with sensitivity to both overt and subclinical epileptic activities.

Methodologically, our PLS‐based framework represents an advance in noninvasive epilepsy mapping.^30^ By extracting a single latent component that maximally covaries with known epileptic activation patterns,^3^, ^13^ the approach harnesses information from dozens of rs‐fMRI features simultaneously, effectively decoding the complex network signature of epilepsy from high‐dimensional data.^31^ In doing so, we reinforce the concept of epilepsy as a network disorder of distributed functional abnormalities rather than isolated focal changes.^49^, ^50^ Unlike prior studies relying on one or few rs‐fMRI metrics or on trained machine‐learning classifiers,^14^, ^15^, ^16^, ^17^, ^18^, ^19^, ^20^, ^21^ this data‐driven technique requires no supervised model training. Instead, it can be applied at the individual patient level, enhancing clinical translatability. By leveraging a static template of epileptic activation rather than real‐time EEG events, this method can infer epileptic network activity even when no spikes are captured, a scenario where conventional EEG‐fMRI would yield no findings. This benefit addresses a major clinical limitation of EEG‐fMRI and highlights the framework's potential in routine, resting‐state scans.^10^ At the same time, TDC did not exhibit spurious “epileptic” PLS1 activation, emphasizing the method's high specificity for true epileptic network patterns. PLS1 is thus suggested as a composite biomarker with superior sensitivity and consistency, relative to any single metric. These attributes make the approach especially promising for clinical applications, as it could identify abnormal brain areas from a single resting‐state scan without the need for prolonged EEG monitoring or patient cooperation during a task.

Biologically, the PLS1 maps and feature weights provide insights that are consistent with known epilepsy network mechanisms. In CAE, PLS1 was most prominent in the thalamus, in agreement with the well‐established role of thalamocortical circuits in generalized 3 Hz spike‐and‐wave seizures.^46^, ^47^, ^48^ In SeLECTS, PLS1 showed maximal involvement of the rolandic region, accurately capturing the syndrome's typical centrotemporal spike focus.^2^, ^11^, ^19^ These syndrome‐specific localizations bolster confidence that PLS1 is detecting genuine pathophysiological substrates. Moreover, the features contributing most strongly to PLS1 differed by syndrome in a biologically meaningful way. In SeLECTS, amplitude‐based metrics (such as ALFF) were among the top weighted features,^22^, ^26^ whereas in CAE, connectivity‐based measures (notably thalamic functional connectivity density) dominated PLS1's composition.^46^, ^47^, ^48^ This divergence echoes known pathophysiological differences: SeLECTS is thought to stem from focal cortical hyperexcitability, whereas childhood absence epilepsy involves hypersynchronized thalamocortical network oscillations.^2^ Thus, beyond improving detection, the PLS model may mechanistically highlight which aspects of brain function are most perturbed in each syndrome. We also observed that PLS1 remained elevated (although at lower magnitude) in the epileptogenic regions during no‐discharge rest sessions compared to discharge sessions and healthy baseline, a pattern of stepwise activation unique to PLS1 and not seen with any individual rs‐fMRI metric. This aggregate result suggests the presence of an ongoing “epileptic predisposition” state in brain networks even in the apparent absence of spiking, consistent with prior reports of enduring network abnormalities in both SeLECTS and CAE patients during interictal periods.^19^, ^48^, ^51^ Our integrated approach may thus capture an association between pediatric epilepsies and persistent functional impairments in specific brain circuits.

Despite these strengths, there are several limitations to this study. First, we did not have a definitive ground truth (e.g., stereo‐electroencephalography or post‐surgical outcomes)^6^, ^7^ to confirm the exact source of epileptic activity; localization was inferred from EEG‐fMRI maps and syndrome‐based clinical definitions. Second, our single‐center pediatric cohort may limit generalizability. Multicenter studies spanning broader ages and scanner platforms are needed. Finally, the framework depends on syndrome‐specific activation priors. Extending our approach will require building a library of reproducible templates for additional epilepsy subtypes through methods such as multicenter EEG‐fMRI pooling. Overall, these steps will be key to translating template‐guided multivariate rs‐fMRI decoding into a robust clinical tool, with potential applicability to other conditions characterized by reproducible network signatures.

## 5 CONCLUSIONS

This study introduces a PLS‐based multivariate rs‐fMRI framework that can localize and quantify epileptic brain activity in children with SeLECTS and CAE. The approach demonstrated robust syndrome‐specific localization of epileptogenic regions, remained sensitive to abnormalities even in the absence of overt interictal discharges, and showed good performance in distinguishing epilepsy types as well as patients from healthy children. These results highlight the potential of multivariate rs‐fMRI integration as a clinically relevant tool for noninvasive epilepsy evaluation.

## AUTHOR CONTRIBUTIONS

Conceptualization: Qirui Zhang, Joseph I Tracy, Zhiqiang Zhang, and Guangming Lu. Resources: Yan He, Fang Yang, and Zhiqiang Zhang. Methodology: Qirui Zhang and Zixuan Zhang. Investigation: Qirui Zhang and Zixuan Zhang. Visualization: Qirui Zhang and Jacqueline Kim. Funding acquisition: Qirui Zhang and Zhiqiang Zhang. Supervision: Guangming Lu and Zhiqiang Zhang. Writing – original draft: Qirui Zhang, Zixuan Zhang, and Jacqueline Kim. Writing – review and editing: Joseph I Tracy and Zhiqiang Zhang.

## CONFLICT OF INTEREST STATEMENT

The authors declare no competing interests. We confirm that we have read the Journal's position on issues involved in ethical publication and affirm that this report is consistent with those guidelines.

## Supporting information


Figure S1.

## Data Availability

The data that support the findings of this study are available from the corresponding author upon reasonable request.
