# Supplementary material for: Integrating multivariate resting‐state fMRI features to localize epileptic networks in common childhood epilepsy
Source: Epilepsia. 2026 May 6;67(7):3550–62. doi: 10.1002/epi.70243 (PMC13361039; doi:10.1002/epi.70243)
Supplement: Supplementary file 1 — Figure S1. [file EPI-67-3550-s001.docx]

1. **Data Acquisition**

Patients were instructed not to fall asleep and to remain awake. Foam pads helped anchor the EEG leads, reduce motion, and improve patient comfort. For EEG recording, FCz was set as the reference, and electrocardiography was recorded using an electrode placed on the back. Functional MRI data were acquired using a T2*-weighted single-shot echo-planar imaging (EPI) sequence with the following parameters: repetition time (TR) = 2000 ms, echo time (TE) = 30 ms, field of view (FOV) = 240 × 240 mm², in-plane matrix = 64 × 64, flip angle = 90°, 30 axial slices, slice thickness = 4 mm with a 0.4-mm interslice gap. High-resolution structural MRI data were acquired in the sagittal plane using a three-dimensional T1-weighted magnetization-prepared rapid gradient-echo (MPRAGE) sequence (TR = 2300 ms, TE = 2.98 ms, flip angle = 9°, FOV = 256 × 256 mm², acquisition matrix = 256 × 256, slice thickness = 1 mm, no interslice gap).

1. **Quality control**

This study enrolled 222 self-limited epilepsy with centrotemporal spikes (SeLECTS) sessions (9.13±2.10 years),130 childhood absence epilepsy(CAE) sessions (9.75±3.48years) and typically developing controls (TDC) (9.42±2.48 years). We conducted quality control of fMRI and sMRI data for these sessions.

In sMRI quality control, one radiologist rated the raw T1WI quantity on a scale 1 to 5 representing image quality from low to high. We exclude subjects with sMRI quality score less than 4 (RE, sub-sessions =10; AE, sub-sessions =8; HC, sub-sessions=0).

In fMRI quality control, one radiologist visual inspected fMRI images for full cortex coverage, artifact, and signal loss. Then preprocessed fMRI data and retain head motion for each sub-session. Finally, the sub-sessions with maximum relative head motion larger than 1.5mm or averaged relative head motion larger than 0.35mm were excluded (RE, sessions =30; AE, sessions =37; HC, sessions=8).


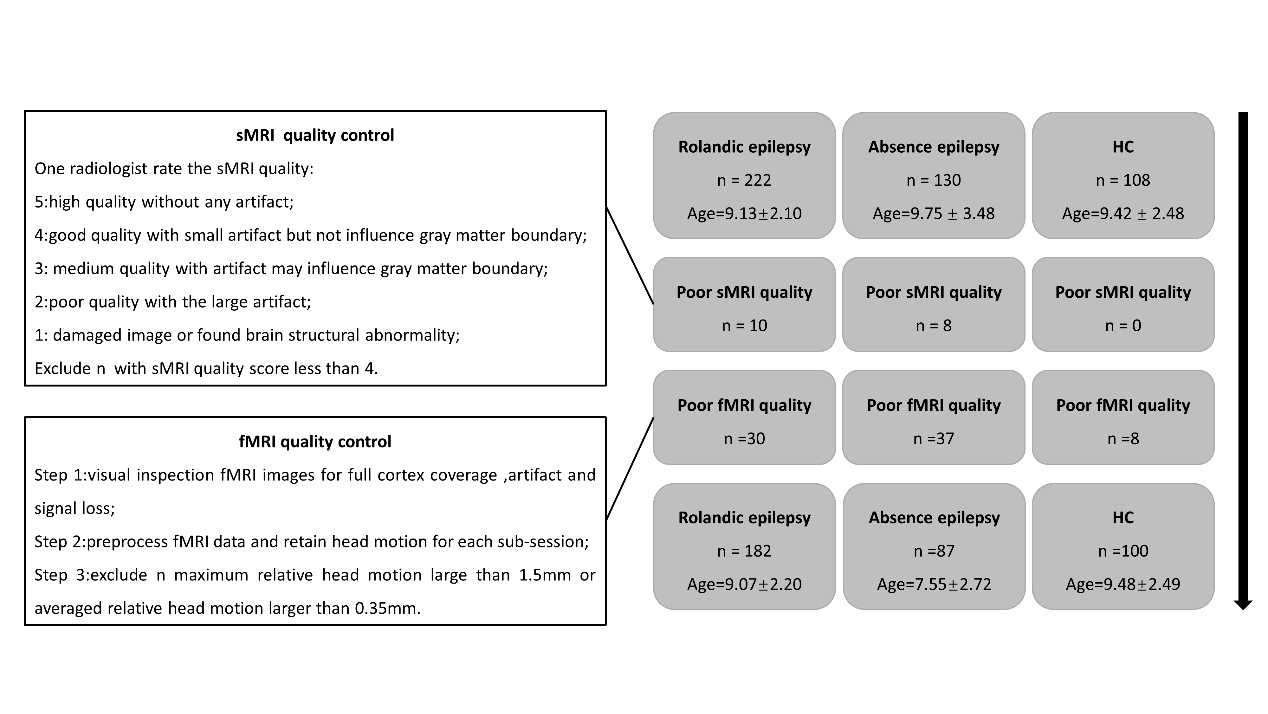
A total of 182 SeLECTS sessions (9.07±2.20 years), 87 CAE sessions (7.75±2.72years) and TDC (9.48±2.49 years) finally included to analysis after quantity control (Figure S1).

**Figure S1. Quality control of imaging data**

1. **The Definition and Computation of Regional rs-fMRI Parameters**

Only the following metrics were additionally computed within four narrow frequency bands (0.01–0.027 Hz, 0.027–0.073 Hz, 0.073–0.198 Hz, and 0.198–0.25 Hz), in addition to the conventional low-frequency band: amplitude of low-frequency fluctuation (ALFF), fractional ALFF (fALFF), regional homogeneity (ReHo), Hurst exponent (Hurst), degree centrality (DC), and long- and short-range functional connectivity density (FCD).
All other metrics, including Granger causality density (GCD), resting-state lag analysis (RSLA), and hemodynamic response function (HRF)–related parameters, were computed using the full-band resting-state signal and were not frequency-band–specific.

Unless otherwise specified, all parameter maps were masked using a gray matter probability mask with a threshold of 0.2 prior to subsequent analyses.

1. Amplitude of Low-Frequency Fluctuation (ALFF) and Fractional ALFF (fALFF)

ALFF quantifies the amplitude of spontaneous low-frequency fluctuations in the BOLD signal. For each voxel, the preprocessed rs-fMRI time series was transformed into the frequency domain using Fourier transformation, and ALFF was defined as the mean amplitude within a specified frequency range.

fALFF is a normalized version of ALFF and was calculated as the ratio between the ALFF within a target frequency band and the total spectral amplitude across the entire measurable frequency range of the BOLD signal. This normalization reduces the contribution of non-specific physiological noise.

Both ALFF and fALFF were computed for the conventional low-frequency band and additionally for each of the four narrow frequency bands.

1. Regional Homogeneity (ReHo)

ReHo measures local synchronization of spontaneous neural activity. It was defined using Kendall’s coefficient of concordance (Kendall’s W) among the time series of a given voxel and those of its nearest neighboring voxels, reflecting local temporal consistency within a functional cluster.

ReHo was computed on unsmoothed data, and the resulting ReHo maps were spatially smoothed afterward. ReHo was calculated for the conventional low-frequency band and additionally for each of the four narrow frequency bands.

1. Hurst Exponent

The Hurst exponent was estimated voxel-wise using in-house implementations based on established fractal analysis methods. Hurst maps were computed for the conventional low-frequency band and additionally for each of the four narrow frequency bands.

1. Degree Centrality (DC)

Degree centrality quantifies the global functional importance of each voxel by measuring its connectivity with the rest of the brain. For each voxel, Pearson correlation coefficients were calculated between its time series and those of all other voxels within a gray matter mask.

An undirected adjacency matrix was constructed by applying a correlation threshold of r > 0.25 to remove weak or noise-driven connections. Weighted degree centrality was then computed as the sum of suprathreshold connections for each voxel.

DC was calculated on unsmoothed data and spatially smoothed afterward. DC maps were generated for the conventional low-frequency band and additionally for each of the four narrow frequency bands.

1. Long- and Short-Range Functional Connectivity Density (FCD)

Functional connectivity density (FCD) measures the density of suprathreshold functional connections associated with each voxel and was subdivided into short-range and long-range components using a neighborhood-based approach.

Short-range FCD was defined as the number of suprathreshold correlations (r > 0.25) between a voxel and other voxels located within a local spherical neighborhood with a radius of 12 mm or less.

Long-range FCD was defined as the number of suprathreshold correlations between the voxel and voxels outside this local neighborhood (radius greater than 12 mm).

Long- and short-range FCD maps were converted to standardized values for statistical analyses and spatially smoothed. Both measures were computed for the conventional low-frequency band and additionally for each of the four narrow frequency bands.

1. Granger Causality Density (GCD)

Granger causality density (GCD) is a directed effective connectivity measure that characterizes voxel-wise causal information flow across the brain. For each voxel, multivariate autoregressive modeling was used to estimate whether past activity in one voxel significantly improved the prediction of future activity in other voxels. Due to the high computational demand of voxel-wise GCD were computed on gray matter–masked fMRI data resampled to 6 × 6 × 6 mm³, consistent with prior studies.

Voxel-wise maps of input (inflow) and output (outflow) causal connectivity were generated by summarizing significant directed influences between the voxel and the rest of the brain. GCD was computed using the full-band resting-state signal and was not frequency-band–specific.

1. Resting-State Lag Analysis (RSLA)

Resting-state lag analysis (RSLA) characterizes the relative temporal delays of spontaneous BOLD signal fluctuations across brain regions. Voxel-wise signal latency was estimated by identifying temporal shifts that maximized similarity between local time series and a reference signal. Due to the high computational demand of voxel-wise RSLA were computed on gray matter–masked fMRI data resampled to 6 × 6 × 6 mm³,

RSLA was computed using the full-band resting-state signal and was not performed separately within narrow frequency bands.

1. Hemodynamic Response Function (HRF)–Related Parameters and HRF-Deconvolved Metrics

HRF-related analyses were performed to characterize regional hemodynamic properties and to reduce the confounding effects of vascular variability on rs-fMRI measures. Voxel-wise HRF parameters included response height, time to peak, and full width at half maximum.

Latent neural time series were further estimated via HRF deconvolution. Based on these deconvolved signals, HRF-based ALFF, fALFF, and degree centrality measures were derived. All HRF-related parameters and HRF-deconvolved metrics were computed using the full-band resting-state signal and were not frequency-band–specific.
